# Side-Directed Release of Differential Extracellular Vesicle-associated microRNA Profiles from Bronchial Epithelial Cells of Healthy and Asthmatic Subjects

**DOI:** 10.3390/biomedicines10030622

**Published:** 2022-03-07

**Authors:** Viktoria E. M. Schindler, Fahd Alhamdan, Christian Preußer, Lukas Hintz, Bilal Alashkar Alhamwe, Andrea Nist, Thorsten Stiewe, Elke Pogge von Strandmann, Daniel P. Potaczek, Clemens Thölken, Holger Garn

**Affiliations:** 1 Translational Inflammation Research Division & Core Facility for Single Cell Multiomics, Philipps University of Marburg–Medical Faculty, Member of the German Center for Lung Research (DZL) and the Universities of Giessen and Marburg Lung Center, 35043 Marburg, Germany; schindlerviktoria@t-online.de (V.E.M.S.); alhamdaf@staff.uni-marburg.de (F.A.); hintz@staff.uni-marburg.de (L.H.); potaczek@staff.uni-marburg.de (D.P.P.); 2 Institute for Tumor Immunology, Philipps University of Marburg–Medical Faculty, 35043 Marburg, Germany; preusserc@staff.uni-marburg.de (C.P.); bilal.alashkaralhamwe@staff.uni-marburg.de (B.A.A.); elke.poggevonstrandmann@uni-marburg.de (E.P.v.S.); 3 Core Facility Extracellular Vesicles, Philipps University of Marburg–Medical Faculty, 35043 Marburg, Germany; 4 College of Pharmacy, International University for Science and Technology (IUST), Daraa 15, Syria; 5 Institute of Molecular Oncology & Genomics Core Facility, Philipps University of Marburg–Medical Faculty, Member of the German Center for Lung Research (DZL) and the Universities of Giessen and Marburg Lung Center, 35043 Marburg, Germany; andrea.nist@imt.uni-marburg.de (A.N.); stiewe@uni-marburg.de (T.S.); 6 Institute of Medical Bioinformatics and Biostatistics, Philipps University of Marburg–Medical Faculty, 35043 Marburg, Germany; thoelken@uni-marburg.de

**Keywords:** bronchial epithelial cells, extracellular vesicles, miRNAs, airway epithelium, asthma, cellular compartmentalization

## Abstract

Extracellular vesicles (EVs) are released by virtually all cells and may serve as intercellular communication structures by transmitting molecules such as proteins, lipids, and nucleic acids between cells. MicroRNAs (miRNAs) are an abundant class of vesicular RNA playing a pivotal role in regulating intracellular processes. In this work, we aimed to characterize vesicular miRNA profiles released in a side-directed manner by bronchial epithelial cells from healthy and asthmatic subjects using an air−liquid interface cell culture model. EVs were isolated from a culture medium collected from either the basolateral or apical cell side of the epithelial cell cultures and characterized by nano-flow cytometry (NanoFCM) and bead-based flow cytometry. EV-associated RNA profiles were assessed by small RNA sequencing and subsequent bioinformatic analyses. Furthermore, miRNA-associated functions and targets were predicted and miRNA network analyses were performed. EVs were released at higher numbers to the apical cell side of the epithelial cells and were considerably smaller in the apical compared to the basolateral compartment. EVs from both compartments showed a differential tetraspanins surface marker expression. Furthermore, 236 miRNAs were differentially expressed depending on the EV secretion side, regardless of the disease phenotype. On the apical cell side, 32 miRNAs were significantly altered in asthmatic versus healthy conditions, while on the basolateral cell side, 23 differentially expressed miRNAs could be detected. Downstream KEGG pathway analysis predicted mTOR and MAPK signaling pathways as potential downstream targets of apically secreted miRNAs. In contrast, miRNAs specifically detected at the basolateral side were associated with processes of T and B cell receptor signaling. The study proves a compartmentalized packaging of EVs by bronchial epithelial cells supposedly associated with site-specific functions of cargo miRNAs, which are considerably affected by disease conditions such as asthma.

## 1. Introduction

Asthma is a common non-communicable inflammatory disease of the airways, affecting more than 339 million people worldwide, and is a major cause of morbidity around the globe [1,2]. Disease pathogenesis in asthma involves the interaction of many different cell types within the respiratory tract, including CD4+ T-cells, granulocytes, dendritic cells, macrophages, myeloid-derived regulatory cells, natural killer cells, smooth muscle cells, and airway epithelial cells [3]. These various types of cells communicate via many different signaling mechanisms, such as soluble factors, including cytokines and chemokines. In the past decade, a new mechanism of intercellular communication by extracellular vesicles (EVs) was discovered [4]. They have been found in all body fluids, including in blood [5], urine [6], and bronchoalveolar lavage fluid (BALF) [7]. All EVs are composed of a lipid bilayer containing transmembrane proteins and can be classified into exosomes, microvesicles, and apoptotic bodies depending on size, structural components, and generation process [8]. While exosomes are approximately 30–150 nm in diameter and are derived from the exocytosis of multivesicular bodies, microvesicles are shed at the cell surface and are 50–1000 nm in size [9,10]. EVs express membrane proteins, which frequently have been used as surface markers to identify specific EV subsets such as exosomes, or to trace the cell of origin. Universally expressed exosomal proteins such as the tetraspanins CD9, CD63, and CD81 are therefore commonly used as exosomal markers to distinguish exosomes from other EV subsets [11], although conflicting data describe the expression on both exosomes and microvesicles [12].

EVs function as intercellular communicators transporting diverse lipids, proteins, and nucleic acids, such as DNA and certain types of RNA including messenger RNA (mRNA) and small RNAs, such as microRNAs (miRNAs), small interfering RNAs (siRNAs), transfer RNAs (tRNAs), and PIWI-associated RNAs (piRNAs) [13]. Small RNAs are less than 200 nucleotides long and are not translated into proteins, but rather regulate biological processes by interfering with mRNA translation. MiRNAs are defined as short non-coding single-stranded RNAs with a length of approximately 22 nucleotides. They target mRNA, inducing mRNA degradation or inhibiting protein translation, and thereby regulate gene expression [14]. Vesicular miRNAs are protected from degradation by RNA-degrading enzymes (RNAses) in body fluids due to the protective shell provided by the vesicles lipid bilayer, and therefore can be shuttled between cells [15].

The airway epithelium is known to account for a variety of abnormal responses in asthma, such as epithelial mucus metaplasia [16,17]. Increasing evidence further suggests an active role of lung epithelial cells in the initiation and perpetuation of local immune mechanisms not only by the secretion of cytokines, but also as a major producer of EVs [18,19,20,21]. Many studies have described a potential proinflammatory role of EVs in allergy and asthma, but with few studies specifically addressing the role of EVs derived from airway epithelial cells. Vesicles secreted by lung epithelial cells have been shown to prime immune cells toward proinflammatory features [22]. Furthermore, a differential expression of extracellular miRNAs in asthmatic patients compared to non-asthmatics with a downstream regulatory impact on inflammation has been described [23,24]. However, to the best of our knowledge, so far, no study has explicitly distinguished between vesicular miRNA profiles on the apical and basolateral cell side of airway epithelial cells.

The purpose of this study was to investigate vesicle characteristics and vesicular miRNA profiles associated with EVs derived from the airway epithelial cells of healthy and asthmatic subjects depending on the cell side of secretion. Therefore, an air−liquid-interface cell culture model of airway epithelial cells was used for sample collection. We then performed small RNA sequencing and conducted extensive bioinformatic analyses to identify vesicular miRNA signatures. Furthermore, miRNA associated roles, functions, and targets were predicted by associated target genes, and miRNA network analysis was conducted to reveal closely related functional clusters within the identified set.

## 2. Materials and Methods

### 2.1. Cell Culture

**Figure 1:**
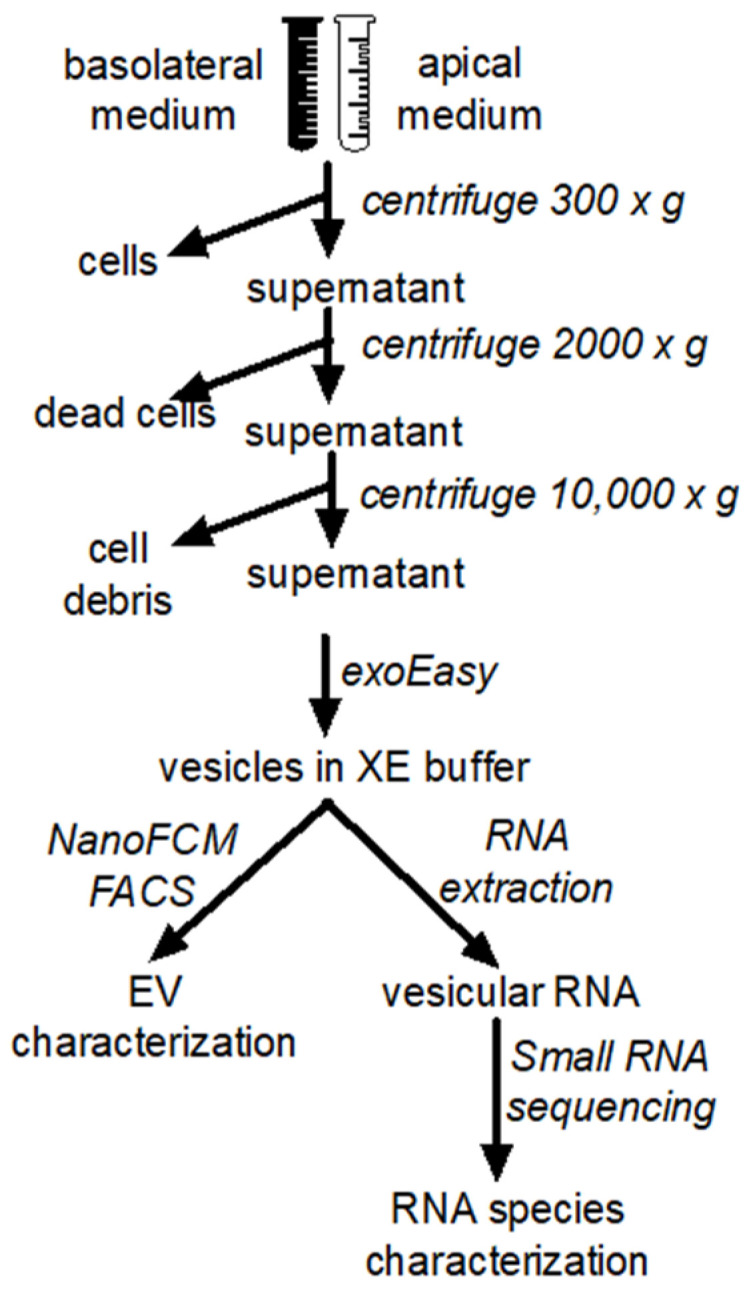
Schematic overview of EV preparation steps.

MucilAir^TM^ primary human bronchial epithelial cells were purchased from Epithelix (Epithelix, Sárl, Geneve, Switzerland) and cultured using air−liquid interface conditions. Cultures were established from three different healthy non-smoking donors (two males and one female, aged 15, 41, and 71 years, respectively) and three different asthmatic non-smoking donors (two males and one female, aged 36, 50, and 55 years, respectively). MucilAir^TM^ cell culture medium (Epithelix) was exchanged and the cells were washed carefully with a medium from the apical side to remove residual mucus on a regular basis, according to the manufacturer’s instructions. The cell culture medium was continuously collected over one month from the basolateral and apical cell sides. While basal samples were directly retrieved from the bottom chamber of the culture system, for collection of the apical samples, the cells were incubated with 200 µL apically applied cell culture medium for 30 min at 37 °C, and afterwards they were washed by carefully pipetting up and down. All samples were stored at −80 °C until further use. The general experimental downstream workflow is shown in Figure 1.

### 2.2. EV Isolation

After thawing at room temperature, conditioned medium samples were sequentially centrifuged at 4 °C to remove cell debris and large vesicles (10 min at 500× *g*, 20 min at 2000× *g* and 30 min at 10,000× *g*). Next, the samples were passed through a 0.22 µm filter (Millex-GS Syringe filter unit, Merck KGaA, Darmstadt, Germany) and the EVs were isolated using the exoEasy kit (Qiagen GmbH, Hilden, Germany) from 4 mL of basolaterally and 600 µL of apically collected conditioned medium, according to the manufacturer’s protocol [25]. 

### 2.3. NanoFCM Analysis

For the Flow Nano Analyzer (NanoFCM Co. Ltd., Nottingham, UK) analysis, the system was calibrated using 200 nm polystyrene beads (NanoFCM Co. Ltd., Nottingham, UK) with a defined concentration of 5.7 × 10^8^ particles/mL, which were also used as a reference for the particle concentration. In addition, monodisperse silica beads (NanoFCM Co. Ltd., Nottingham, UK) of four different sizes were used as reference standards to calibrate the size of the EVs. Freshly filtered (0.22 µm) PBS was analyzed as a background signal, which was subtracted from the other measurements. EV samples were diluted with filtered PBS resulting in a particle count in an optimum range of 2500–12,000 events, and sample data were collected for 1 min with a sample pressure of 0.4 kPa. Particle concentration and size distribution were calculated using the NanoFCM software NF Profession v1.08) (NanoFCM Co. Ltd., Nottingham, UK). Median and interquartile range (IQR) were calculated using R software v4.1.0 (R Foundation, Vienna, Austria).

### 2.4. Total Protein Quantification

The EV sample protein concentration measurement was performed on NanoDrop^TM^ (Thermo Fisher Scientific, Waltham, MA, USA) at 280 nm, with an estimated percent extinction coefficient of 10. The analysis was performed in triplicate on non-diluted EV isolates.

### 2.5. Bead-Based Flow Cytometry

EVs were detected by bead-based flow cytometry as previously described by Benedikter et al., with some adaptations [26]. Briefly, 4 µm aldehyde/sulphate latex beads at 3.5 × 10^8^/mL (Thermo Fisher Scientific, Waltham, MA, USA) were incubated with 0.125 mg/mL of an equal mixture of three monoclonal antibodies (anti-human CD9 (clone M-L13), anti-human CD63 (clone H5C6), anti-human CD81 (clone JS-81); all antibodies were purchased from BD Biosciences, Franklin Lakes, NJ, USA) overnight in an MES buffer (Sigma Aldrich, St. Louis, MO, USA) on a shaker at 6500 rpm. The coated beads were stored in PBS containing 0.1% (*m*/*v*) glycine and 0.1% (*m*/*v*) sodium azide at 4 °C until use. Before use, the beads were washed with PBS containing 2% (*w*/*v*) bovine serum albumin (BSA). Then, 1 × 10^6^ beads in 1 µL were incubated overnight with 100 μL of isolated EVs suspended in PBS at a concentration of 1.6 × 10^7^ particles/mL based on NanoFCM measurements. Detection was performed as described previously, with either one or a mixture of the following phycoerythrin (PE)-labelled antibodies: anti-human CD9, anti-human CD63, and anti-human CD81 (same clones as above) [26]. Stained beads were suspended in 150 μL PBS and were analyzed using a BD FACSCanto II and FACS Diva v8.0.1 analysis software (BD Biosciences, Franklin Lakes, NJ, USA). To quantify the EV surface marker expression, relative fluorescence units (RFU) were calculated by multiplying the percentage of PE-positive beads with the median fluorescent intensity (MFI) of the positive bead population, as described by Benedikter et al. [26]. 

### 2.6. Statistical Analysis of Numerical Data

Data were analyzed using GraphPad Prism v7 (GraphPad Software Inc., San Diego, CA, USA) using Student’s unpaired t-test for concentration and size distribution, and the Whitney−Mann U-test for FACS analysis with *p*-values * < 0.05, ** < 0.01, *** < 0.001, and **** < 0.0001. Data are presented as mean ± SEM.

### 2.7. Vesicular RNA Extraction

RNA extraction was performed from 200 µL of EV solution using the miRNeasy kit (Qiagen GmbH, Hilden, Germany), according to the manufacturer’s instructions. The RNA concentration was assessed using the Qubit™ microRNA Assay Kit (Thermo Fisher Scientific, Waltham, MA, USA). RNA size distribution and yield were analyzed using the Agilent 2100 Bioanalyzer with the Small RNA analysis kit (Agilent Technologies, Santa Clara, CA, USA).

### 2.8. Small RNA Sequencing

Small RNA libraries were constructed using NEBNext Small RNA Library Prep Set for Illumina (New England Biolabs, Ipswich, MA, USA), according to the manufacturer’s protocol, with minor modifications for the low RNA input. Briefly, 3 ng of RNA was used for the library preparation. The 3′ SR Adapter, SR RT Primer, and 5′ SR Adapter were diluted 1:4, and the RNA was ligated with both adapters, and was reverse transcribed, barcoded, and amplified for 15 cycles. The generated libraries were cleaned up using AMPure XP Beads (Beckman Coulter, Brea, CA, USA) and quantified using the Qubit™ dsDNA HS Assay (Thermo Fisher Scientific, Waltham, MA, USA) and the Bioanalyzer High Sensitivity DNA Analysis kit (Agilent Technologies) prior to sequencing on a NextSeq550 platform (Illumina, San Diego, CA, USA) with High Output Kit v2.5 and 50 bases single-reads, according to the manufacturer’s instructions. 

### 2.9. Bioinformatic Analysis

The reads were first trimmed for the first three nucleotides (-u 3) and adapters overlapping at least five nucleotides with the read (-O 5) using cutadapt v2.9 [27]. Reads shorter than 15 nucleotides were discarded (-m 15). Trimmed reads were mapped using bwa-mem v0.7.17-r1188 in three steps, with the minimum score output (-T) and seed length (-k) set to 15 [28]. The reads were mapped successively against a custom list of transcripts containing ribosomal RNAs (rRNAs) from rFam 14.1, mature miRNAs from miRbase 22.1, ncRNAs from ENSEMBL release 97, piRNAs from piRNA-DB v1.7.5, and cDNAs from ENSEMBL release 97 as the references. In this order, unmapped reads from each step were mapped against the next reference to assure unique attribution per RNA type. The reads were counted per transcript with Samtools v1.10 [29], and 374 miRNAs with more than 10 reads across all samples were analyzed using R package DESeq2 v1.28.1 for the differential gene expression [30]. Differences were classified as significant with a threshold of absolute value of fold change (FC) > 2 and FDR < 0.05. Principal component analysis was conducted and visualized using R package pcaExplorer v2.14.2 based on variance stabilized transformed read counts of miRNAs [31]. Heatmaps were generated using R package pheatmap v1.0.12 with default clustering parameters. KEGG (Kyoto Encyclopedia of Genes and Genomes) pathway analyses were performed using DIANA-miRPath v3.0 with a FDR threshold < 0.05 and other default settings [32]. miRNAs function, family, disease, and regulatory proteins were analyzed using TAM 2.0 by masking cancer-related terms and keeping the other default settings [33]. Network analysis was conducted by miRTargetLink Human with strong evidence, and the resulting genes were uploaded on STRING v11.0 for the Reactome pathways [34].

## 3. Results

### 3.1. EVs Secreted by Airway Epithelial Cells Are Mainly Released at the Apical Cell Surface

**Figure 2:**
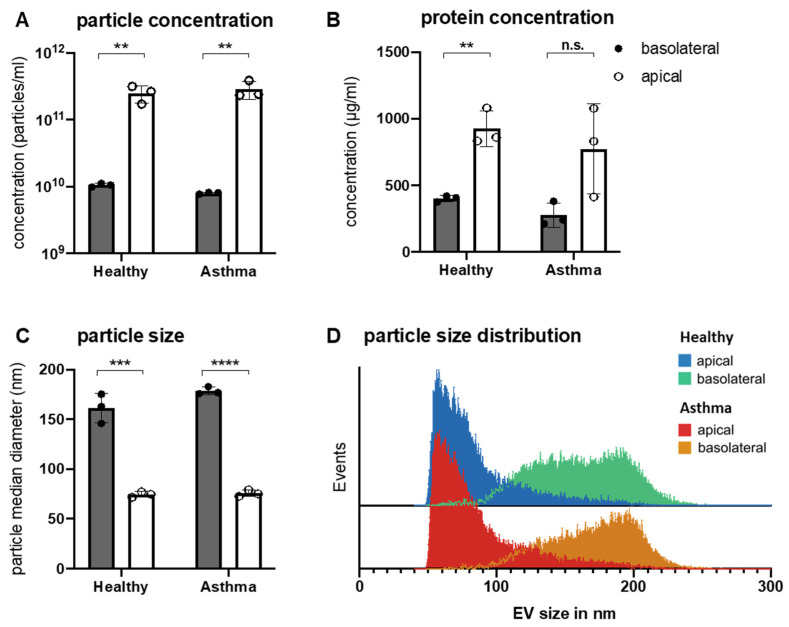
EV characteristics in apically and basolaterally secreted vesicles. (**A**), Particle concentration after EV isolation analyzed by NanoFCM. (**B**), Protein concentration in EV isolates measured by NanoDrop Protein A280. (**C**), Median particle diameter and (**D**) size distribution of EVs isolated from the apical or basolateral side of bronchial epithelial cell cultures from healthy and asthmatic subjects analyzed by NanoFCM. Bars represent mean ± SD; dots indicate individual samples. ** *p* < 0.01, *** *p* < 0.001, **** *p* < 0.0001. EV—extracellular vesicle; n.s.—not significant.

To confirm that the isolated particles were indeed EVs, and to compare numbers and composition of apically versus basolaterally released EVs, NanoFCM analysis and bead-based flow cytometry with staining for characteristic tetraspanin surface markers of EVs were performed. Particle concentrations and size profiles showed significant differences in NanoFCM analysis, depending on their isolation side. Generally, epithelial cells secreted significantly larger quantities of particles at the apical cell surface compared to the basolateral side. NanoFCM analysis showed higher particle concentrations in apically obtained EV samples (Figure 2A). This finding was further confirmed by the results of protein concentration analyses, as depicted in Figure 2B. Furthermore, the size of particles retrieved from the apical cell side wash corresponded to the typical size range of exosomes with a mean median size of 75 nm (62–95 nm). Contrarily, particles isolated from the basolateral side were considerably larger in size with a mean median size of 169 nm (140–192 nm), consistent in size with microvesicles rather than with exosomes (Figure 2C,D). No differences in concentration or size range were observed between particles from healthy and asthmatic subjects.

### 3.2. Differential Surface Marker Expression Profiles between Apically and Basolaterally Released EVs

**Figure 3:**
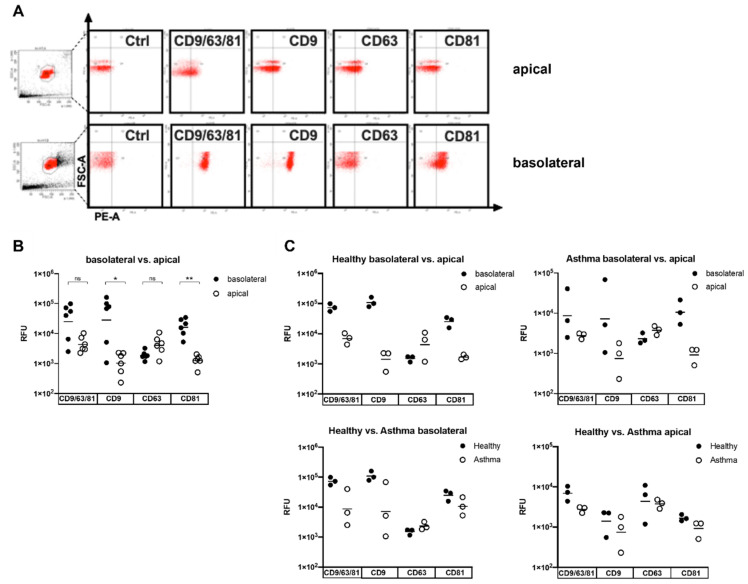
Bead-based flow cytometry analysis of tetraspanins surface marker expression on EVs released by airway epithelial cells. Vesicles were captured using beads coated with a mixture of CD9-, CD63-, and CD81-specific antibodies. Specific secondary antibodies coupled with either CD9, CD63, or CD81, or a combination of all three, were used for detection. (**A**) The results of one representative experiment demonstrating tetraspanin expression on EVs isolated from apical and basolateral cell culture medium are shown. (**B**) Tetraspanin expression in EVs isolated from basolateral versus apical cell culture medium regardless of disease expression. (**C**) Comparison of the surface marker expression of apical and basolateral EVs in healthy and asthmatic subjects. n = 3 in each group, * *p* < 0.05, ** *p* < 0.01. EV—extracellular vesicle; ns—not significant; RFU—relative fluorescent units.

Bead-based flow cytometry using cocktails of antibodies directed towards the EV-identifying tetraspanin surface markers CD9, CD63, and CD81 for bead binding and detection did not reveal any significant differences between apical and basolateral EVs. However, when examining the expression of singular surface proteins, vesicles secreted to the basolateral side showed a higher expression of CD9 and CD81 compared to apically secreted vesicles, while for CD63, such a difference could not be observed (Figure 3A,B). When further looking at the subgroups, namely EVs from asthmatic and healthy subjects, these differences in marker expression depending on secretion side were similarly represented. However, CD9 expression by apical compared to basolateral EVs seemed to differ to a greater extent in the EVs of healthy subjects. When comparing the surface marker expression of EVs from healthy and asthmatic subjects, subtle distinctions in the expression of CD9 were observed on the basolateral side, but not for the other tetraspanins. No differences could be found in these groups for vesicles from the apical cell side (Figure 3C). This differential representation of selective marker proteins on EVs was generally suggestive for compositional differences, depending on the cell side of secretion.

### 3.3. Apically and Basolaterally Released EVs Show Distinct RNA Cargo Composition

Small RNAs were isolated from EVs retrieved from the apical and basolateral compartments of airway epithelial air−liquid interface cultures from healthy and asthmatic subjects (each n = 3), and were further analyzed by small RNA sequencing. All libraries exhibited a minimum of 7.5 million uniquely mapped reads and were thus comparable in efficiency. RNA composition was determined by counting the percentages of reads mapped to different species of RNA, such as rRNAs, long-non-coding RNAs (lncRNAs), miRNAs, mRNAs, and piRNAs. Our analysis showed a significant difference in the composition of RNA subtypes between apical and basolateral EV populations. Apical EVs contained comparable proportions of miRNAs (37.3%) and lncRNAs (33.3%) as te most prominent fractions, while basolateral EVs contained miRNAs only at a percentage of 2.3% and were rather dominated by a high percentage of lncRNAs (50.5%; Supplementary Figure S1).

**Figure 4:**
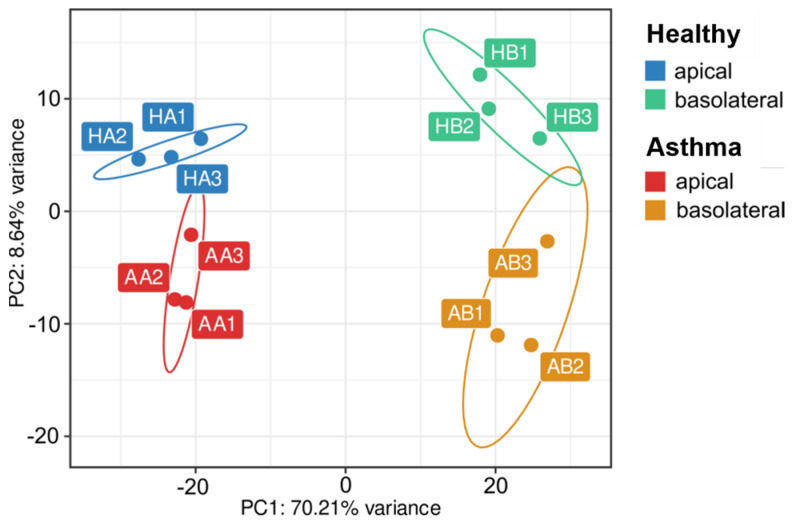
PCA plot depicting the clustering of 12 EV samples according to their miRNA cargo depending on disease condition and cellular side of EV release. PCA—principal component analysis; EV—extracellular vesicle.

**Figure 5:**
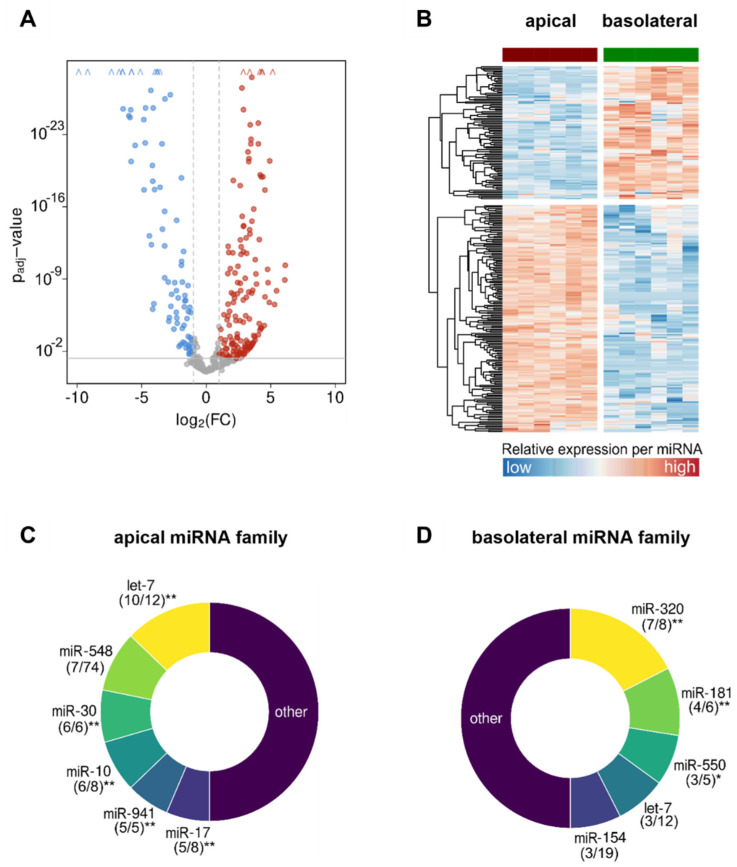
Differential expression analysis of vesicular miRNAs in apical versus basolateral compartments of bronchial epithelial cell cultures. (**A**,**B**) Volcano plot and heat map showing the differentially expressed miRNAs within EVs secreted to the apical versus basolateral compartment at *p*_adj_ < 0.05 and FC ≥ 2, and (**C**,**D**) donut charts showing the distribution of the mainly represented miRNA families in both compartments. Digits in brackets depict the number of enriched miRNA family members out of the total number of miRNAs belonging to the respective family, * *p* < 0.05, ** *p* < 0.01, miRNA—microRNA; EV—extracellular vesicle; FC—fold change.

Further focusing on miRNAs composition, apical and basolateral vesicles could be clearly separated from each other as two distinct populations in a principal component analysis. Moreover, in each of these populations, two clearly different clusters representing either the healthy or the asthmatic condition were clearly distinguishable (Figure 4). When looking at differences in miRNAs composition between apical and basolateral EVs in all 12 samples, we found 236 significantly differentially expressed miRNAs between the two subgroups, of which 151 miRNAs were more and 85 miRNAs less abundant in the apical compared to the basolateral EVs (Figure 5A,B). More frequent miRNAs in the apical population were assigned to different miRNA families (groups of miRNAs with a high sequence similarity deriving from distinct genomic loci) than those found at higher levels in the basolateral EVs (Figure 5C,D). In apically secreted EVs, all family members of the miR-30 (6/6) and the miR-941 (5/5) family were present, pointing to a significant association of these miRNAs to processes specifically important to the apical environment. Additionally, 10 out of 12 miRNAs from the let-7, 6 out of 8 of the miR-10, and 5 out of 8 of the miR-17 families were present. On the basolateral side, the most represented miRNA family was the miR-320 family with 7 out of 8 members, followed by the miR-181 (4/6), the miR-550 (3/5), the let-7 (3/12), and the miR-154 (3/19) families.

**Figure 6:**
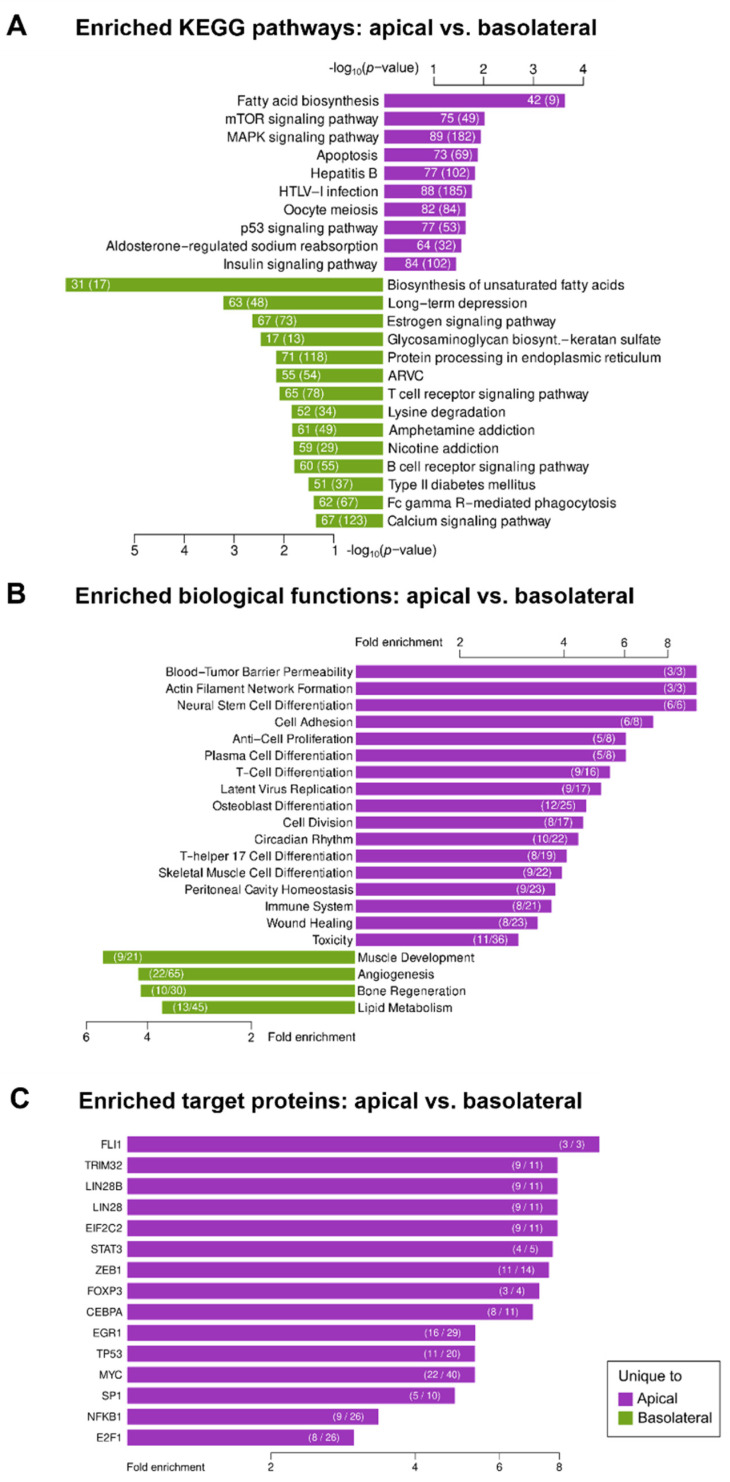
Functional analysis of miRNAs differentially expressed in EVs released by bronchial epithelial cells to the apical versus basolateral compartments. (**A**) KEGG pathway and (**B**) biological functions analyses of differentially expressed miRNAs in both compartments and (**C**) target proteins potentially regulated by the differentially expressed miRNAs. EV—extracellular vesicle.

We then investigated whether these differentially distributed EV miRNAs could be linked to specific biological effects by evaluating the KEGG pathways and biological functions predicted to be affected by them, according to the two databases, DIANA-miRPath v3.0 and TAM 2.0. As shown in Figure 6, the significantly associated KEGG pathways of the preferentially apically secreted miRNAs included, among others, the mTOR and MAPK signaling pathways. Interestingly, miRNAs on the basolateral side were associated with processes of T and B cell receptor signaling, along with others (Figure 6A). Thus, associated KEGG pathways deviated in apically and basolaterally secreted EVs, suggesting different downstream functions for EVs depending on the site of action that are linked to diverse potential biological functions, as shown in Figure 6B. Significantly enriched target regulatory proteins can be found in Figure 6C. The results showed very distinct differences in miRNA composition as well as in downstream targeted proteins and pathways of vesicular RNA, depending on their cell side of secretion.

### 3.4. Small RNA Cargo Is Altered in Vesicles from Asthmatic Subjects

**Figure 7:**
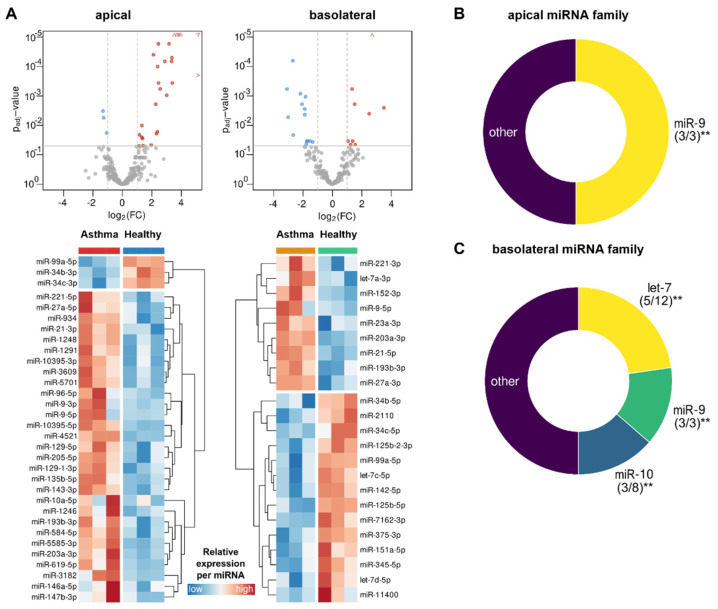
Differential miRNA expression analysis per disease condition (asthma versus healthy) and compartment (apical and basolateral). (**A**) Volcano plot and heat map of differentially expressed miRNAs of asthma versus healthy comparison in EVs from the apical (left) and basolateral (right) cell culture compartments at *p*_adj_ < 0.05 and FC ≥ 2, and (**B**,**C**) donut charts showing the distribution of the mainly represented miRNA families in the asthma versus healthy comparison in both compartments. Digits in brackets depict the number of enriched miRNA family members out of total number of miRNAs belonging to the respective family, ** *p* < 0.01. miRNA—microRNA; EV—extracellular vesicle; FC—fold change.

We additionally checked whether EVs derived from epithelial cells of healthy and asthmatic subjects differed in their miRNA expression profiles regardless of the secretion side, and found overall 12 miRNAs differentially represented in the two groups, with 6 being up- and 6 down-regulated in the vesicles of asthmatics (Supplementary Figure S2). A more complex picture was obtained when additionally taking the EV secretion side into account (Figure 7A). In apical EVs, 32 miRNAs showed a significant difference in abundances in the two groups, 29 of which were up- and 3 of which were down-regulated in the vesicles of asthmatics (Figure 6A, left). On the basolateral side, 23 miRNAs with a divergent expression profile were detected, 9 being significantly upregulated and 14 being downregulated in asthmatics (Figure 7A, right), with 5 out of 12 being family members of the let-7 family and with 3 out of 8 being members of the miR-10 family (Figure 6C). Specifically, the miR-9 family showed significant differences between the healthy and the asthmatic phenotype origin in both EV secretion compartments (Figure 7B,C). The KEGG pathway terms and biological functions associated with differentially abundant miRNAs in apical and basolateral EVs from asthmatic versus healthy subject’s bronchial epithelial cell cultures are shown in Supplementary Figure S3A,B, respectively. Enriched associated diseases included a variety of inflammatory conditions, among them asthma, especially when the analysis was based on the signals from the basolateral side (Supplementary Figure S3C). Significantly enriched target regulatory proteins in asthmatic subjects were HIF1A and NFKB1 (Supplementary Figure S3D). The target genes of the EV-derived miRNAs differentially expressed between both conditions are shown in Supplementary Figure S4A,B, associated with some of the most significant pathways.

## 4. Discussion

In the past decade, the role of EVs as communication structures between neighboring or remote cells has been increasingly recognized. The presence of EV-associated RNAs has been attested by next-generation sequencing in numerous body fluids, including blood plasma and sputum [35]. Specific miRNA signatures hold the potential for being used as fingerprints, helping to identify phenotypes or states of diseases and gain more insights into their underlying pathological mechanisms. Plenty of studies have analyzed miRNA profiles in patients with asthma compared to subjects not affected by this disease, some with a specific focus on the role of airway epithelial cells in EV-related miRNA generation [36]. However, to date, no investigations have explicitly distinguished between EV RNA profiles in polarized airway epithelial cells depending on their direction of secretion. Hence, no distinction has been made between their supposed location of action being either the outer epithelial environment, e.g., sputum, or compartments within the body, such as lung interstitium, tissue, or even blood plasma.

According to the results of the NanoFCM particle characterization, EV populations isolated from the apical cell side were mainly composed of vesicles with diameters matching the size range of the exosomes. Contrarily, on the basolateral cell side, median vesicle diameters were noticeably larger, consistent rather with the size of the microvesicles than with the exosomes [37,38]. FACS analysis for exosomal marker proteins revealed the presence of exosomes in both apical and basolateral samples, although apical EVs seemed to express exosomal marker proteins to a greater extent than the basolateral vesicles. 

In our study, we were able to observe distinct differences in EV-associated miRNA patterns secreted by bronchial epithelial cells from healthy and asthmatic subjects, depending on the side of EV secretion. Interestingly, secretion patterns of EV miRNAs varied more distinctly based on the side of secretion than the pathophysiological condition. This knowledge might be essential for future investigations into potential biomarkers analyzed in different compartments such as sputum and plasma. Bartel et al. recently published a PCR-based study comparing the expression of specific miRNAs in EVs secreted by normal human bronchial epithelial cells to the basolateral and apical cell side. Interestingly, there were some notable overlaps in miRNAs, including miR-34b and miR-21 preferentially identified on the apical side, while other differentially expressed miRNAs in this study could not be observed in our analysis [39]. Notably, serum levels of miR-21 have been previously reported to be an efficient biomarker for asthma patients [40]. Further, treatment with a miR-21-specific antagomir was demonstrated to reduce airway hyperresponsiveness and restore steroid sensitivity in mice with ovalbumin-induced allergic airway inflammation [41]. The differentially expressed miR-10 on the apical side was found to regulate the proliferation of airway smooth muscle cells by suppressing the phosphoinositide 3-kinase (PI3K) pathway [42]. Moreover, MAPK and mTOR signaling pathways were enriched as potential targets for differentially expressed miRNAs on the apical side. Activation of the mTOR pathway has been shown to lead to tight junction susceptibility and epithelial–mesenchymal transition (EMT), which can in turn play an essential role in airway remolding in asthma pathogenesis [43,44]. Accordingly, inhibition of MAPK signaling pathway led to a significant reduction in the allergic inflammation of the airways [45]. In contrast, miR-221, which was downregulated on the basolateral side, was shown to play a unique role in controlling the differentiation of Th17 and regulatory T (Treg) cells through targeting SOCS-1 (suppressor of cytokine signaling 1) [46]. Likewise, T and B cell receptor signaling pathways have been associated with miRNAs dominantly secreted to the basolateral side. These pathways have been intensively investigated as key regulators of the antigen recognition in the adaptive immune response and have been utilized as key therapy targets in asthmatic patients [47,48]. Taken together, our observations strongly indicate a compartmentalized packaging and side-specific release of EVs by bronchial epithelial cells, pointing to site-specific functions of these structures at least partially mediated by their miRNA cargo.

While differences in miRNA profiles from apical versus basolateral sides were more pronounced, a number of significant alterations in the levels of certain EV-associated miRNAs (or their families) were also observed between cells of an asthmatic or healthy origin, either at one or at both sides. For example, EV miRNAs belonging to the miR-9 family were upregulated in both apical and basolateral vesicles of asthmatic patients. Increased levels of miR-9 have already been linked to steroid-resistant and neutrophilic, but not eosinophilic asthma [49]. Interestingly, Bazzoni et al. observed a miR-9-dependent inhibition of NFκB1 transcription in human neutrophils and monocytes exposed to proinflammatory signals, suggesting that the rapid induction of miR-9 operates as a feedback control of NFκB1-dependent cellular response [50]. Accordingly, NFκB1 was found among the predicted targets of miRNAs, specifically present in asthmatic samples in our investigation. Several independent studies further identified enhanced NFκB-pathway activation in asthmatic tissue [51]. 

Interestingly enough, miR-34b and miR-34c were downregulated when associated with EVs released by epithelial cells from asthmatic subjects at both compartments, even though they were generated from opposite DNA strands, dependent on the compartment. The levels of miR-34b/c have been found to be significantly lowered in murine mouse models of ovalbumin-induced allergic airway inflammation and have been suggested to play a regulatory role in the activation of the Nrf2-/ARE pathway [52]. Disruption of Nrf2-expression augmented airway inflammation and hyperresponsiveness [53,54,55]. Moreover, in a clinical study by Solberg et al., treatment with corticosteroids resulted in increased levels of miR-34b/c in BALF of patients suffering from asthma, while the administration of IL-13 was able to repress its expression in an air−liquid interface bronchial epithelial cell culture model [56]. This suggests a protective role of miR-34b/c against allergic and asthmatic cellular responses, and hints toward a diagnostic potential of the miR-34 family.

Generally, miRNA of the let-7 family showed a higher expression in apical compared to basolateral EVs. However, in basolateral EVs, the let-7 family was the miRNA subgroup with the largest divergence between asthmatic and healthy subjects, with 5 out of 12 total family members showing significant alterations, while on the apical side, no differences in expression were found. In contrast to this finding, Levänen et al. described significant variations in 16 miRNA, including the let-7 and miR-200 families and miR-99 (as seen in our study) in BALF [24]. Interestingly, while let-7c and let-7d were downregulated, let-7a showed an upregulation in EVs released by cells from asthmatics. miRNAs of the let-7 family are among the most intensely studied miRNAs, with two studies proposing the exosome-mediated transfer of let-7 miRNAs to various immune cells as a suppressive mechanism used by Treg cells (let-7d), and reporting the inhibition of Treg cell generation and function by these miRNAs (let-7i) [57,58].

There are some limitations to this study that should be mentioned. One limitation is the small sample size, yet the major goal of the study was a general overview of EV fingerprints in different cellular compartments, rather than assessing the function of singular EV miRNAs. For a more detailed analysis, further studies investigating the role of single miRNA are required to give the data a clinical significance. Another limitation is that there was no information available about the clinical details and/or the asthmatic phenotype of the patients whose cells were involved in the study. Lastly, as miRNAs can be secreted by almost every cell type, the overall in vivo situation can largely dissociate from in vitro observed conditions. Dissecting the contribution of individual cell types in the production of specific miRNA is, on the other hand, essential to understand their role in disease pathogenesis.

In summary, in this study, we provide a general overview of miRNA-composition in EVs secreted by airway epithelial cells. We were able to reveal distinctly differing miRNA expression profiles depending on the vesicle side of secretion and disease condition. This emphasizes the importance of taking the vesicle site of action into consideration for further research to which the data presented in this study provide a sound basis.

## Data Availability

The data presented in this study and underlying raw data are available on reasonable request from the corresponding author.

## Supplementary Materials

The following supporting information can be downloaded at: https://www.mdpi.com/article/10.3390/biomedicines10030622/s1. Supplementary Figure S1: Comparison of EV total RNA composition between basolateral and apical compartments regardless of disease expression in percentage of reads mapped to indicated RNA species. EV—extracellular vesicle; rRNA—ribosomal RNA; mRNA—messenger RNA; lncRNAs—long-non-coding RNAs; piRNA—PIWI-associated RNAs; miRNA—microRNA. Supplementary Figure S2: Differential miRNA expression analysis per disease condition (asthma versus healthy) regardless of compartment (apical and basolateral). (A) Volcano plot and (B) heat map exhibiting the differently expressed miRNAs of the asthma versus healthy comparison at *p*_adj_ < 0.05 and FC ≥ 2. miRNA—microRNA; FC—fold change. Supplementary Figure S3: Functional analysis of the differentially expressed miRNAs in the asthma versus healthy comparison in each apical and basolateral compartment. (A) KEGG pathway and (B) biological function analyses of differentially expressed vesicular miRNAs of asthma versus healthy in each apical and basolateral compartment, (C) enriched diseases, and (D) target proteins potentially regulated by the differentially expressed miRNAs. miRNA—microRNA. Supplementary Figure S4: mRNA−miRNA network analysis involving differentially regulated miRNAs of the asthma versus healthy comparison in (A) the apical and (B) the basolateral compartments associated with biological pathways of potential target genes. miRNA—microRNA.
