# Supplementary material for: Side-Directed Release of Differential Extracellular Vesicle-associated microRNA Profiles from Bronchial Epithelial Cells of Healthy and Asthmatic Subjects
Source: Biomedicines. 2022 Mar 7;10(3):622. doi: 10.3390/biomedicines10030622 (PMC8945885; doi:10.3390/biomedicines10030622)
Supplement: Supplementary file 1 [file biomedicines-10-00622-s001.zip › biomedicines-1620954-supplementary.pdf]

Supplementary Figure S1

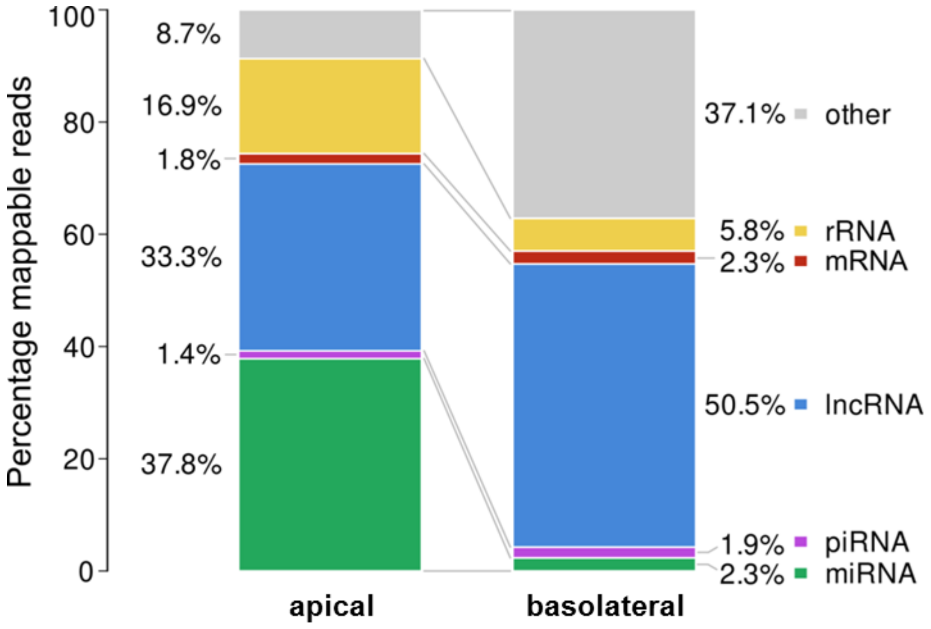

Supplementary Figure S1. Comparison of EV total RNA composition between basolateral and apical compartments regardless of disease expression in percentage of reads mapped to indicated RNA species. EV—extracellular vesicle; rRNA—ribosomal RNA; mRNA—messenger RNA; lncRNAs—long-non-coding RNAs; piRNA—PIWI-associated RNAs; miRNA—microRNA.

Supplementary Figure S2

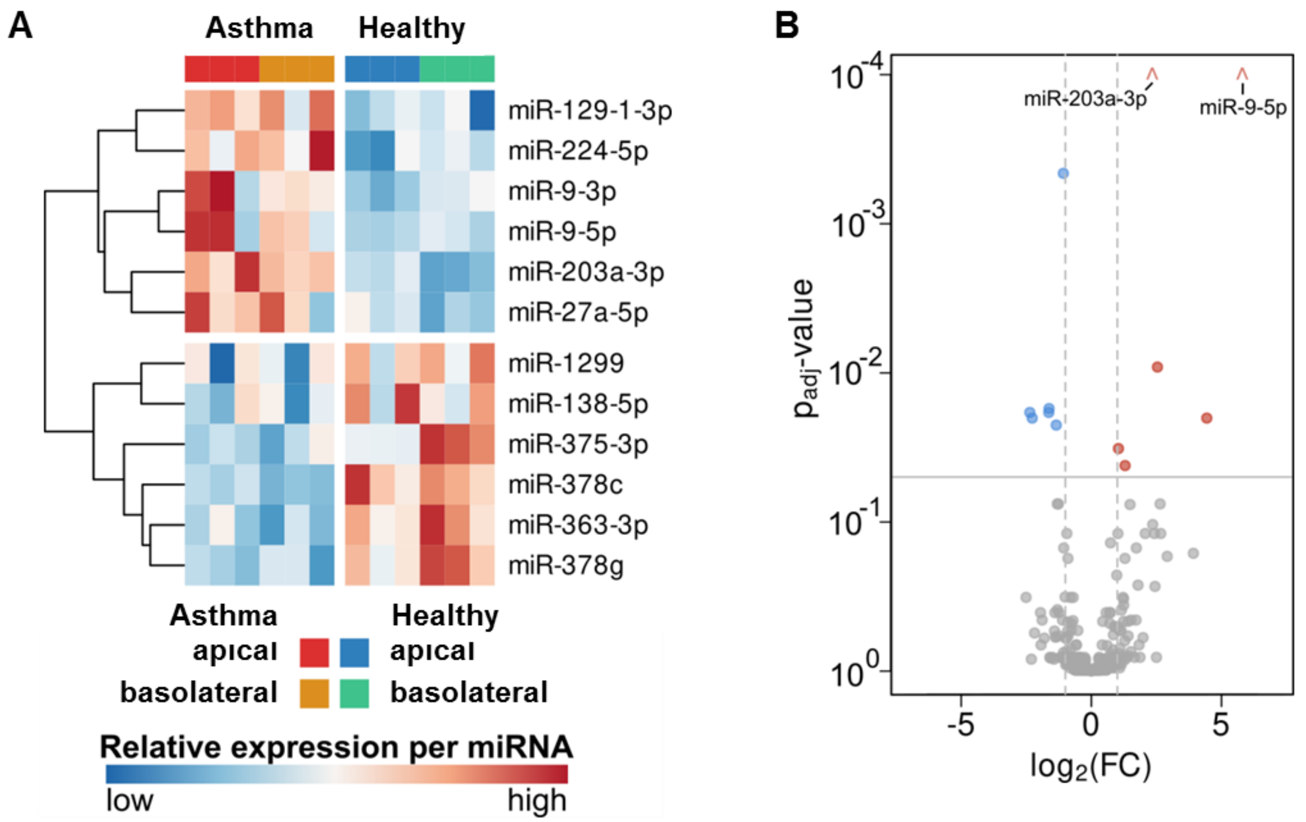

Supplementary Figure S2. Differential miRNA expression analysis per disease condition (asthma versus healthy) regardless of compartment (apical and basolateral). (A) Volcano plot and (B) heat map exhibiting the differently expressed miRNAs of the asthma versus healthy comparison at $p_{adj} < 0.05$ and FC ≥ 2. miRNA—microRNA; FC—fold change.

Supplementary Figure S3A & B

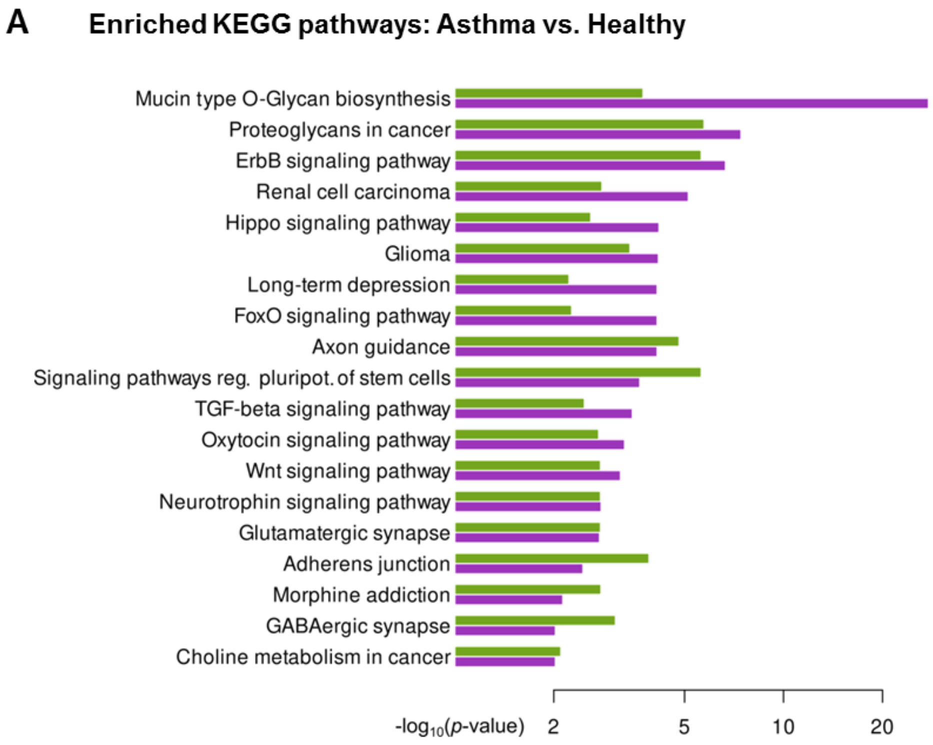

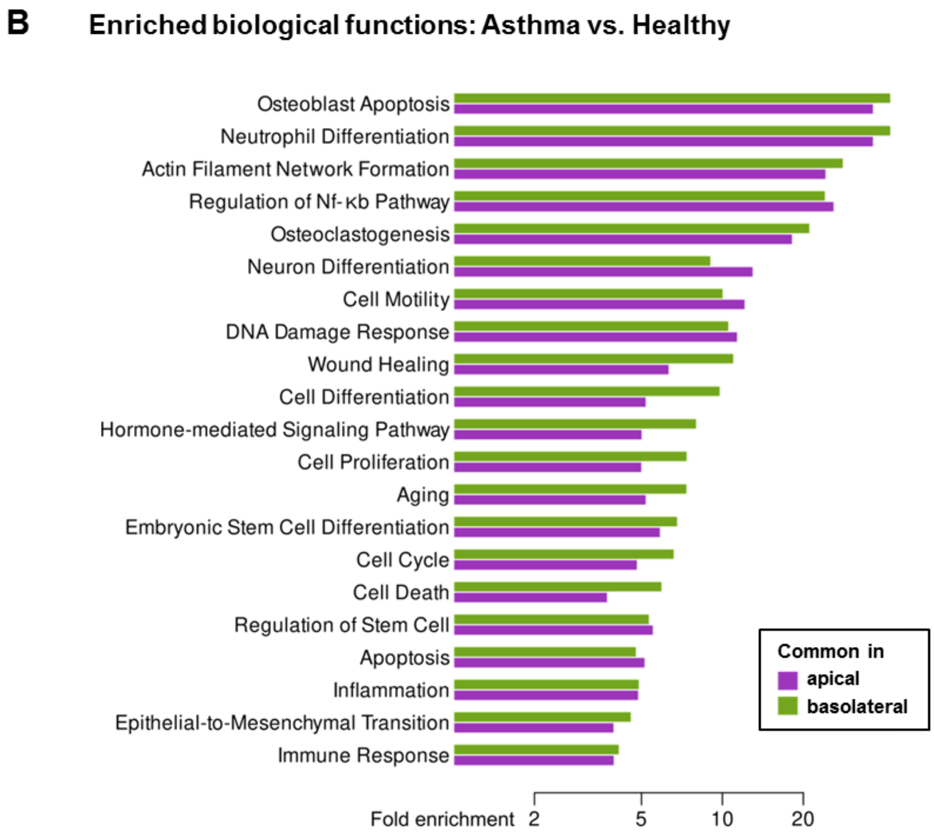

Supplementary Figure S3C & D

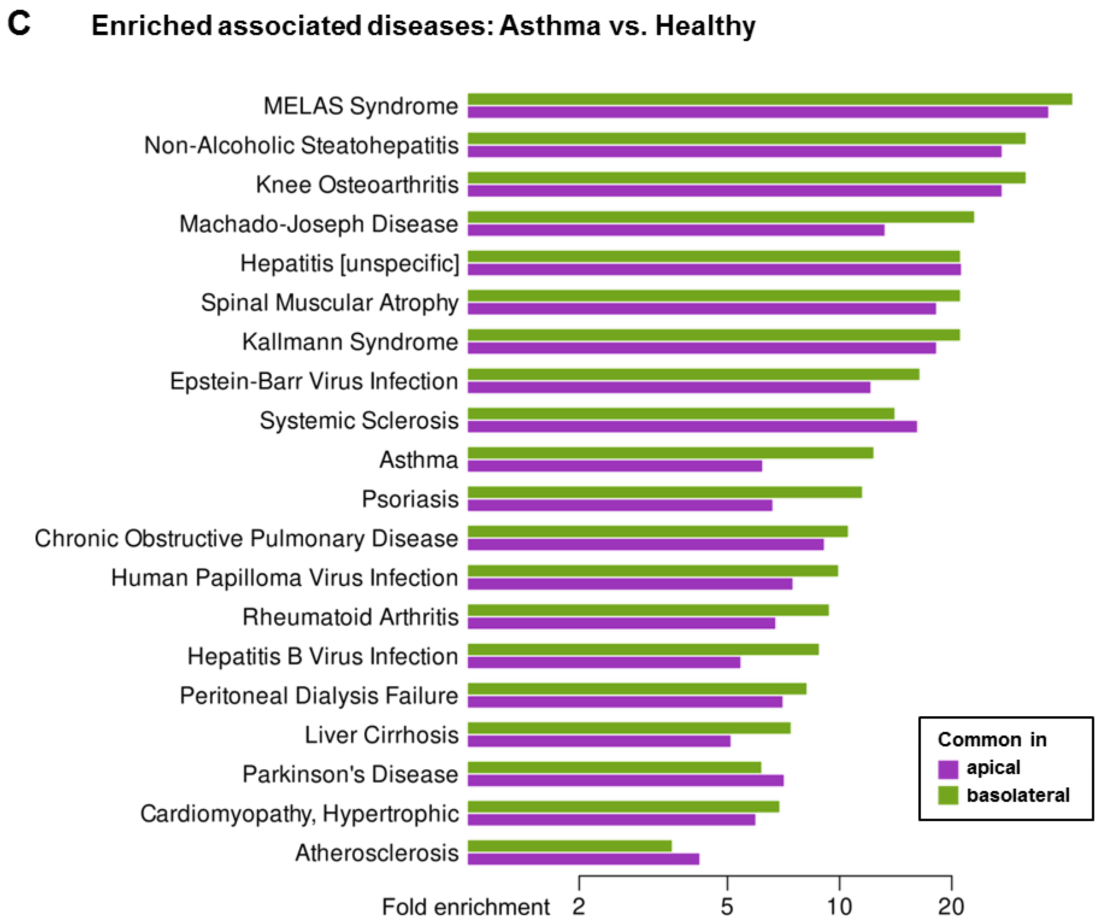

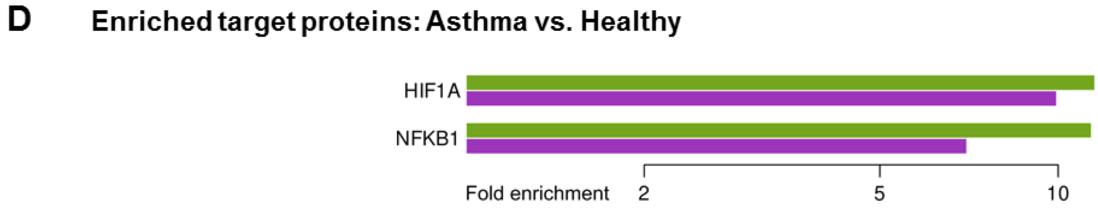

Supplementary Figure S3. Functional analysis of the differentially expressed miRNAs in the asthma versus healthy comparison in each apical and basolateral compartment. (A) KEGG pathway and (B) biological function analyses of differentially expressed vesicular miRNAs of asthma versus healthy in each apical and basolateral compartment, (C) enriched diseases, and (D) target proteins potentially regulated by the differentially expressed miRNAs. miRNA—microRNA.

Supplementary Figure S4A

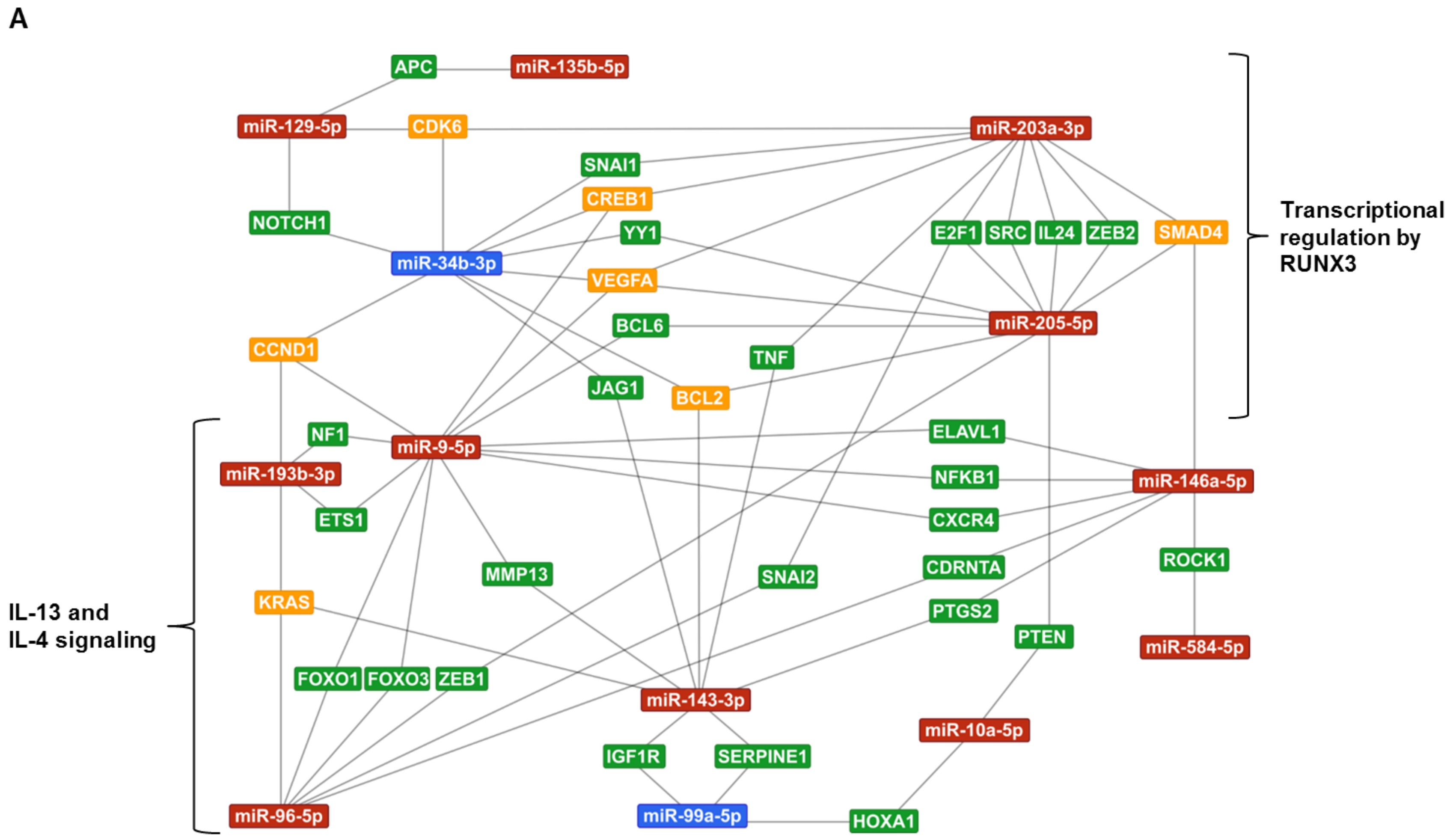

Supplementary Figure S4B

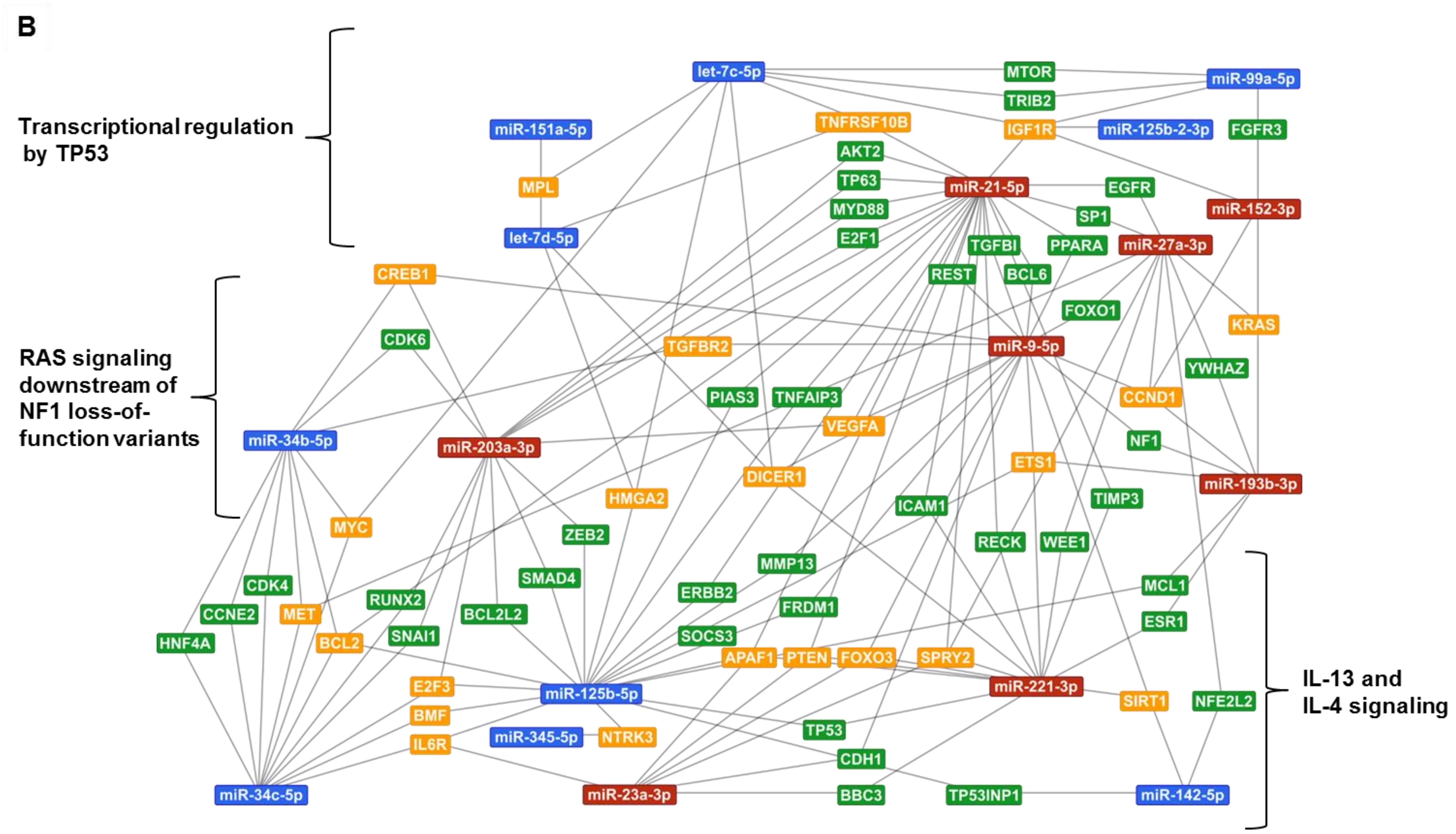

Supplementary Figure S4. mRNA−miRNA network analysis involving differentially regulated miRNAs of the asthma versus healthy comparison in (A) the apical and (B) the basolateral compartments associated with biological pathways of potential target genes. miRNA—microRNA.
